# Help‐seeking behaviour in newly diagnosed lung cancer patients: Assessing the role of perceived stigma

**DOI:** 10.1002/pon.4779

**Published:** 2018-07-03

**Authors:** Shiho Rose, Allison Boyes, Brian Kelly, Martine Cox, Kerrin Palazzi, Christine Paul

**Affiliations:** ^1^ Priority Research Centre for Health Behaviour, School of Medicine and Public Health University of Newcastle Callaghan NSW Australia; ^2^ Hunter Medical Research Institute New Lambton NSW Australia; ^3^ Centre for Brain and Mental Health Research, School of Medicine and Public Health University of Newcastle Callaghan NSW Australia; ^4^ Clinical Research, Information Technology and Statistical Support Unit Hunter Medical Research Institute New Lambton NSW Australia

**Keywords:** group identification, help‐seeking, lung cancer, oncology, perceived legitimacy, social support, stigma, support services

## Abstract

**Objective:**

This study explored help‐seeking behaviours, group identification, and perceived legitimacy of discrimination, and its potential relationship with perceived lung cancer stigma.

**Methods:**

Consecutive consenting adults (*n* = 274) with a primary diagnosis of lung cancer within the previous 4 months were recruited at 31 outpatient clinics in Australia. A self‐report survey assessed help‐seeking, group identification, perceived legitimacy of discrimination, and perceived lung cancer stigma.

**Results:**

Services providing assistance from health professionals (69.5%) and informational support (68.5%) were more frequently used than emotional‐based support. Only a small proportion (2.6%) of participants were unlikely to seek help from anyone, with the most popular sources of help being the general practitioner (91.0%), and oncologist/treating clinician (81.3%). One‐fifth (21.1%) indicated they identified with being a lung cancer patient, and most did not perceive discrimination against lung cancer patients. Higher perceived lung cancer stigma was significantly associated with greater perceived legitimacy of discrimination (*P* < 0.001), but not help‐seeking behaviours or group identification.

**Conclusions:**

The relationship between lung cancer stigma and perceived legitimacy of discrimination may guide initiatives to reduce stigma for patients. It is encouraging that perceived stigma did not appear to inhibit help‐seeking behaviours. However, further research in this emerging field is needed to investigate patterns of perceived stigma and help‐seeking over time to identify how and when to offer support services most appropriate to the needs of lung cancer patients.

## 1 BACKGROUND

Lung cancer patients under‐utilise referrals to supportive care services and under‐represent the client profile of community cancer support services.^1^, ^2^, ^3^ This is despite high levels of anxiety and depression^4^ and unmet supportive care needs.^5^ A potential barrier to help‐seeking is the possible stigma associated with lung cancer. For instance, lung cancer stigma has been significantly associated with delays in seeking medical assistance for symptoms,^6^ and deters patients from participating in support groups^7^, ^8^ and discussing their diagnosis with others.^9^

Health‐related stigma is defined as an adverse social judgement *“based on an enduring feature of identity conferred by a health problem or health‐related condition”*.^10^ This negative assessment can be perceived (ie, the anticipation or fear of being discriminated against) or enacted (ie, actual discrimination on account of their condition). These feelings can be internalised by affected individuals to manifest as guilt, shame, and self‐blame.^9^ Given the known links between smoking and disease onset, lung cancer stigma is driven by the belief that the individual's diagnosis was caused through their own behaviours.

People with lung cancer may feel their identity is defined by their diagnosis. Social Identity Theory suggest individuals strive for positive self‐concept, facilitated through their group identity.^11^ The effect of stigma within this paradigm can vary across individuals depending on their resilience in the face of stigma—that is, whether an individual's identity is threatened by their diagnosis and how this may impact their sense of self.^12^ This response links with the extent of their group identification and the perceived legitimacy of discrimination associated with their group.^13^ Individuals may allow their group identity (in this instance being a lung cancer patient) to be internalised as part of their self‐concept, thereby becoming more vulnerable to the stereotypes associated with lung cancer and predict their response to stigma.^14^, ^15^ As such, if an individual perceives discrimination towards their group identity as being fair, they are more likely to have low self‐esteem.^13^ If they perceive the discrimination to be unfair, they may react with feelings of empowerment if they have high group identification, or feelings of indifference if they have low group identification .^13^

Lung cancer patients report feeling isolated, socially withdrawn, and distressed on account of the stigma of their diagnosis.^9^, ^16^ However, it is not known whether group identity and the perceived legitimacy of discrimination play a role in lung cancer stigma and the potential influence on help‐seeking behaviours. Given the evidence to date, further investigation regarding the potential impacts of lung cancer stigma is warranted. The aims of this study were to measure in a sample of patients recently diagnosed with lung cancer:

1. Help‐seeking from support services (ie, interest in and/or use of support service for cancer‐related assistance);
2. Help‐seeking from people (ie, likelihood to approach people for cancer‐related assistance);
3. Group identity (ie, personal identification with being a lung cancer patient or not);
4. Perceived legitimacy of discrimination (ie, belief that perceptions of lung cancer are fair);
5. The relationships between perceived lung cancer stigma and each of help seeking, group identity, and perceived legitimacy of discrimination.

It is hypothesised that participants with greater perceived lung cancer stigma will report lower levels of help‐seeking from services and from people after controlling for age, gender, smoking status, and social support; and that these relationships will be mediated by group identification and perceived legitimacy of discrimination.

## 2 METHODS

This prospective cross‐sectional study was conducted within the baseline phase of a randomised controlled trial investigating online and telephone delivered psychosocial support for people newly diagnosed with lung cancer. As per the protocol of the main trial,^17^ adults who received a primary diagnosis of lung cancer within the last 4 months, were proficient in English, and had some form or internet access were recruited from outpatient oncology and respiratory clinics between September 2014 and December 2016. Consenting patients completed a self‐report questionnaire, with up to 2 reminders at 2 weekly intervals to non‐responders.

Approval was obtained from the Human Research Ethics Committees of: Epworth HealthCare (647‐14); Greenslopes Health (15/46); Hunter New England Local Health District (HREC/14/HNE/168); St John of God (912); Uniting HealthCare (15/29); University of Newcastle (H‐2014‐0690); and University of Tasmania (H0014301). Informed consent was obtained from all individual participants included in the study.

### 2.1 Measures

Where appropriate measures for lung cancer populations were not available, study‐specific items were developed by the authors using an iterative process. Existing measures assessing concepts of interest were examined (predominantly from mental health fields). Draft items were pilot tested with volunteers sourced from a research register to seek their opinions about: clarity and sensitivity of instructions and items; completeness of response options; and format and length. Items were revised based on the feedback received. The final survey comprised the following self‐reported measures:

#### 2.1.1 Help‐seeking from support services

An author‐developed item was used, where a list of 13 support services was presented including emotional (eg, support groups); informational (eg, brochures); health professionals (eg, Cancer Care Coordinator); and practical (eg, financial, legal). Responses were categorised as “I know of this service and I used it”, “I know of this service and I did not use it”, “I do not know of this service and I might like to use it”, and “I do not know of this service and I do not want to use it”. The number of services the participant had indicated either use of (ie, response option “I know of this service and I used it”) or interest in using (ie, response option “I do not know of this service but I might like to use it”) were summated to give a total score (possible range, 0‐13) for analysis. Higher scores indicate greater use or interest.

#### 2.1.2 Help‐seeking from people

The General Help‐Seeking Questionnaire^18^ was adapted, asking “If you were having problems as a result of your lung cancer, how likely is it that you would seek help (personal, emotional or practical help) from any of the following people?” The list of people included: partner; other relative/family; friend/neighbour; oncologist/treating clinician; other health care professional; local general practitioner; and someone else. The original measure was developed for mental health, and for this study options were modified to be more relevant for lung cancer patients (eg, from “mental health professional” to “my oncologist/treating clinician”). Responses were given on a 7‐point Likert scale (“extremely unlikely” to “extremely likely”) and summated to give a total score (possible range, 7‐49). Higher scores indicate greater help‐seeking.

#### 2.1.3 Group identification

An author‐developed item assessed whether participants identified with other lung cancer patients as a group. The item asked “How do you see yourself?” The response options were: “a lung cancer patient (the type of cancer I have been diagnosed with is important)”, “a (general) cancer patient (I am no different from persons with any other form of cancer)”, “as an everyday person with an illness (I am just like anyone else who has an illness of any kind)”, “I do not see myself as a person with an illness”, or “none of the above”. Responses were grouped as either “a lung cancer patient” or “not a lung cancer patient” for analysis.

#### 2.1.4 Perceived legitimacy of discrimination

Two author‐developed items were used: (1) “Generally, I feel people are more sympathetic to persons with other cancer types compared with lung cancer”; and (2) “Generally, I feel that people have unfair views towards persons with lung cancer”. Responses were given on a 4‐point Likert scale (“strongly disagree” to “strongly agree”). A total score was calculated (possible range, 2‐8) for analysis. Higher scores indicated greater perceived legitimacy of discrimination.

#### 2.1.5 Social support

The 12‐item version of the Medical Outcomes Study—Social Support Survey^19^ was included as a potential confounding factor to help‐seeking. Responses are given on a 5‐point Likert scale (“none of the time” to “all of the time”) and calculated to give a total score (possible range, 12‐60). Higher scores indicate greater perceived social support. The abbreviated version has sound reliability, with a Cronbach's α of 0.94.^19^

#### 2.1.6 Lung cancer stigma

The 31‐item Cataldo Lung Cancer Stigma Scale (CLCSS)^20^ measured perceived lung cancer stigma. Responses are given on a 4‐point Likert scale (“strongly disagree” to “strongly agree”) and calculated to give a total score (possible score, 31‐124). Higher scores indicate greater perceived stigma. The CLCSS is a valid and reliable measure for lung cancer patients, with a Cronbach's α of 0.96.^20^

### 2.2 Sample size

The outcome for the variable help‐seeking from support services was number of services, which had an assumed mean of approximately 2. The outcome for the variable help‐seeking from people was behaviour, a continuous measure using a Likert scale (ranging from 1 to 7), which had an assumed mean of 4 and standard deviation of 2. Three‐hundred subjects gave 80% power to detect a 26% poorer help‐seeking from support services among those with an above average level of stigma to those whose level of stigma is below the average at the 5% significance level; and 80% power to detect a 0.16 increase in poorer help‐seeking from people associated with each 10 unit increase in stigma using a 5% level of significance. These were assumed to indicate medium effect sizes.^21^

### 2.3 Statistical analysis

Stata (StataCorp LP, College Station, TX, USA) v14.1 was used for all statistical analyses. Descriptive statistics were used to describe participant characteristics, help‐seeking behaviours (support services and people), group identification, and perceived legitimacy of discrimination.

Quantile regression was used to examine the associations between lung cancer stigma, help‐seeking behaviours, and perceived legitimacy of discrimination (as normality of residuals was not met for linear regression modelling). Binary logistic regression was used to examine the association between lung cancer stigma and group identification. The proposed associations with lung cancer stigma were then examined adjusting for potential confounders chosen a priori based on clinical knowledge and previous literature (age, gender, smoking status, and social support). In the instance where more than 10% of responses from the CLCSS were missing, the case was removed from analysis. Additionally, as heteroscedasticity of residuals was seen, robust standard errors were used to generate 95% CI.

Finally, after adjusting for possible confounders, if a significant association was seen between lung cancer stigma and help‐seeking behaviours, mediation of these relationships through group identification and perceived legitimacy of discrimination were then explored using the methodology by Preacher and Hayes.^22^

## 3 RESULTS

### 3.1 Sample characteristics

Four‐hundred and one patients from 31 outpatient clinics were identified as eligible, with 351 consenting to participate (28 did not respond to the invitation, and 22 declined to participate). Of this, 274 completed the survey (68.3% overall response rate). There were no significant differences between those who did and did not consent to participate in terms of gender. Table 1 describes participant characteristics. Table S1 describes participant's self‐reported clinical characteristics.

**Table 1:** Self‐reported demographic characteristics (*n* = 274)^a^

	Mean ± SD	Range
Age (years)	67.3 ± 8.9	37 – 87
	n (%)	
Gender		
Male	159 (58.0%)	
Female	115 (42.0%)	
Marital status		
Married or defacto	185 (68.0%)	
Widowed, divorced, separated, or never married	87 (32.0%)	
Education		
≤ High school	164 (61.2%)	
Diploma or trade certificate	73 (27.2%)	
Bachelor or postgraduate degree	31 (11.6%)	
Employment		
Currently employed (full‐time, part‐time, on leave)	67 (25.0%)	
Retired or pensioner	151 (56.3%)	
Not working (unemployed, home duties)	37 (13.8%)	
Other	13 (4.9%)	
Smoking status		
Current	28 (10.3%)	
Former	202 (74.3%)	
Never	42 (15.4%)	

a Number of observations varies due to missing data.

### 3.2 Help‐seeking from support services

Of the 13 services listed, participants were aware of an average 7.8 (SD = 4.9) services, had used 2.5 (SD = 2.4) of the services, and indicated interested in a further 3.3 (SD = 4.0) services which were not previously known to them. Table 2 describes participant responses. Table S2 provides further details on participant's awareness, use, and interest for 1 or more support services.

**Table 2:** Participants' reported awareness, use, and interest per support service (*n* = 274)^a^

	I Know of this Service and …		I Do Not Know of this Service and …	
	I Used it	I Did Not Use it	I Might Like to Use it	I Do Not Want to Use it
	*n* (%)	*n* (%)	*n* (%)	*n* (%)
Emotional				
Hospital or clinic counselling services	52 (19.9%)	120 (46.0%)	77 (29.5%)	12 (4.6%)
Other counselling services for cancer patients	30 (11.5%)	126 (48.5%)	90 (34.6%)	14 (5.4%)
Support groups for cancer patients (telephone or face‐to‐face)	17 (6.5%)	136 (52.1%)	89 (34.1%)	19 (7.3%)
Informational				
Fact sheets, brochures, pamphlets, or DVDs about lung cancer	135 (52.3%)	47 (18.2%)	65 (25.2%)	11 (4.3%)
Health professional				
Help from a cancer care coordinator or cancer nurse	123 (46.8%)	57 (21.7%)	76 (28.9%)	7 (2.6%)
Other health professionals (eg, social worker, occupational therapist)	61 (23.3%)	109 (41.6%)	77 (29.4%)	15 (5.7%)
Practical				
Practical assistance (eg, home care services, physical aids)	39 (15.7%)	135 (54.2%)	63 (25.3%)	12 (4.8%)
Financial or financial planning assistance	17 (6.5%)	128 (48.7%)	60 (22.8%)	58 (22.0%)
Legal advice	16 (6.1%)	127 (48.3%)	56 (21.3%)	64 (24.3%)
Help with getting work entitlements	20 (7.9%)	121 (47.6%)	36 (14.2%)	77 (30.3%)
Housing assistance during treatment (eg, isolated patient travel and accommodation scheme)	27 (10.5%)	122 (47.5%)	43 (16.7%)	65 (25.3%)
Assistance with travel to treatment	38 (14.5%)	116 (44.3%)	72 (27.5%)	36 (13.7%)
Parking assistance	83 (31.2%)	83 (31.2%)	83 (31.2%)	17 (6.4%)

a Number of observations varies due to missing data.

### 3.3 Help‐seeking from people

A mean General Help‐Seeking Questionnaire score of 35.6 (SD = 7.9) was reported. Participants indicated that they were more inclined to seek help from their general practitioner, followed by their oncologist/treating clinician (Table S3). Only 2.6% (*n* = 7) indicated that they would not seek help from anyone if they were having problems related to their lung cancer.

### 3.4 Group identity

The majority of participants indicated they did not identify with being a lung cancer patient, with 21.1% reporting they identified with this group. Most identified with being an everyday person with an illness (37.8%) or as a person with cancer (36.2%). A very small proportion did not identify as being a person with an illness (2.8%), while less did not identify with any of the groups (2.0%).

### 3.5 Perceived legitimacy of discrimination

Most participants disagreed or strongly disagreed that people were less sympathetic to lung compared with other cancers (71.5%). Similarly, many disagreed or strongly disagreed that people had unfair views towards lung cancer patients (63.9%).

### 3.6 Associations between perceived lung cancer stigma with outcomes

Perceived lung cancer stigma was shown to have a significant relationship with perceived legitimacy of discrimination (*P* < 0.001; Table 3). When controlling for selected covariates, the associations between perceived lung cancer stigma and help‐seeking from both support services and from people remained non‐significant (*P* = 0.698 and *P* = 0.344, respectively; Table 3). Therefore, mediation via group identification and perceived legitimacy of discrimination was not tested in both models.

**Table 3:** Associations between perceived lung cancer stigma, help‐seeking behaviours, group identification, and perceived legitimacy of discrimination

	Crude			Adjusted^a^		
	β	95% CI	*P*‐Value	β	95% CI	*P*‐Value
Perceived lung cancer stigma						
Help‐seeking from services	0.031	−0.015, 0.078	0.187	0.010	−0.039, 0.058	0.698
Help‐seeking from people	0.059	−0.131, −0.013	0.110	−0.038	−0.116, 0.041	0.344
Group identification	−0.017	−0.037, 0.003	0.096	−0.020	−0.043, 0.002	0.078
Perceived legitimacy of discrimination	0.036	0.024, 0.047	<0.001^b^	0.029	0.019, 0.040	<0.001^b^

a Controlled for age, gender, smoking status, and social support.

b Statistically significant, *P* < 0.05.

## 4 DISCUSSION

To the authors' knowledge, this study is one of the first to examine lung cancer patients' help‐seeking behaviours, group identity, and perceived legitimacy of discrimination, and their potential associations with perceived stigma. Participants reported on average that they have used or would be interested in using less than half of the support services listed. This finding is similar to that of previous studies.^23^, ^24^ Participants mostly endorsed interest in assistance from health professionals (eg, Cancer Care Coordinator or Cancer Nurse). This aligns with their indicated help‐seeking from people, where local general practitioners and oncologists/treating clinicians were identified as the most likely sources of assistance. Other studies demonstrate that patients view health care professionals as the preferred and trusted source of information.^25^, ^26^ Given this, health professionals need to be aware of their pivotal support role and regularly inform patients the variety of support services, and how to identify or access relevant services.

Emotional support services were least used by our sample. While consistent with psychological service use reported in other studies,^2^, ^8^ it is surprising as lung cancer patients have reportedly high rates of anxiety and depression following diagnosis.^4^ Although reasons for low support service use were not explored, this discrepancy may be attributable to a number of factors. One such factor is the perceived usefulness or appeal of certain services. Interviews with a small sample of lung cancer patients revealed that services such as telephone counselling or cancer support groups are not accessed due to cautiousness in opening up with “strangers” and lack of perceived benefits.^27^ Perceptions of psychosocial care can be a barrier, with stigma known to influence mental health services use.^28^, ^29^ Services need to address these attitudes in order to reach patients who may require and best benefit from their support.^30^ Timing is another factor, with evidence suggesting that psychological distress in lung cancer intensifies over time as symptoms worsen and the disease advances.^31^ During the earlier stages of diagnosis patients may be more interested in having a greater understanding of the disease, treatment options, or impact on daily living activities, with emotional services therefore seen to have less relevance at this time.^26^ Finally, potential out‐of‐pocket costs may limit some patients from using services. Although Australia has a government‐funded universal health care system which provides complete or partial subsidies for health care services, some patients experience ongoing financial burden for their cancer‐related care^32^ which may influence decisions to access psychosocial support services.

A significant relationship was found between perceived lung cancer stigma and perceived legitimacy of discrimination. This relationship with stigma has also been found in other mental health studies.^33^, ^34^ Awareness and agreement of lung cancer stereotypes may be linked to the level of perceived stigmatisation experienced by individuals. The messages lung cancer patients receive, either directly from social networks and consultations with health professionals or broadly from the media can resonate and influence how they view themselves. A large population study found respondents attributed greater personal responsibility towards lung cancer patients compared with other cancer groups, and indicated they were more likely to avoid them.^35^ Patients have also noted experiencing negative encounters with health care providers^16^ and find anti‐smoking campaigns to be distressing and encourage victim blaming.^9^ Regular exposures to these events may reinforce that patients are responsible for their diagnosis and negative responses are warranted. Initiatives are needed to change community perceptions in order to address the stigma of lung cancer as well as increase public knowledge to ensure patients are not judged for their condition.

An important finding was that perceived lung cancer stigma was not significantly associated with help‐seeking behaviours. While the evidence is limited, this was unexpected when considering previous research showing lung cancer stigma as a predictor for delays in medical help‐seeking^6^ and lower social support.^20^ Other studies in mental health have also found that stigma predicts service use.^36^ Our finding challenges what is understood about the role of lung cancer stigma in patients and may reflect the recent efforts to raise the public profile of lung cancer.^37^ These results are encouraging, as it suggests perceived stigma may not be a barrier to seeking support for patients with a recent diagnosis. The benefits of social support in lung cancer have been documented^38^ and encouraging positive social networks soon after a diagnosis may be key to supporting patients. Initiatives around providing support for lung cancer could be directed towards groups of people that will have close and continued contact with patients such as their family, friends, or health professionals to facilitate positive relationships and encourage use of services to meet supportive care needs.

### 4.1 Study limitations

Firstly, this study was cross‐sectional and causality cannot be determined. Secondly, the sample comprised predominantly English‐speaking participants diagnosed within the previous 4 months and had internet access, restricting the generalisability of findings across broader cultural and lung cancer populations. Thirdly, in the absence of existing measures, items relating to help‐seeking from services, group identification, and perceived legitimacy of discrimination were developed by the authors. While this was guided by the literature and piloted, the psychometric properties have not been tested. Fourthly, the estimated sample size was not achieved and as such it may be that the study did not have sufficient power to detect a difference. Finally, the potential risk of sample bias should be acknowledged as it is possible that more optimistic patients were selectively invited to be involved. However, attempts to minimise this were addressed by approaching consecutive patients who met eligibility.

### 4.2 Clinical implications

Despite the limitations, this study has several clinical implications. The absence of a relationship between lung cancer stigma and help‐seeking behaviours prompts the question of whether perceived stigma is a barrier to patients soon after diagnosis. However, the relationship between lung cancer stigma and perceived legitimacy of discrimination can provide an insight into initiatives that could reduce stigma and provide support to patients. It may be that perceptions of lung cancer need to be addressed and challenged directly (such as through targeted campaigns) to reduce the effects of stigma. Understanding why patients do not use or are not interested in support services could guide how to provide best care and offer services most appropriate to their need in accordance to their cancer journey. While the results are reassuring for health care providers, further investigations in this field are warranted to consolidate our understanding of this phenomenon over the course of cancer continuum, particularly when lung cancer stigma has been shown to be correlated to other psychosocial and clinical outcomes.

## CONFLICT OF INTEREST

The authors have no conflict of interest to disclose.

## Supporting information


**Table S1** Self‐reported lung cancer characteristics (n=274)*.
**Table S2.** Participants reported awareness, use and interest of one or more support service (n=274).
**Table S3.** Participants’ reported likelihood of seeking help from people (n=274)*.
