# Supplementary material for: Help‐seeking behaviour in newly diagnosed lung cancer patients: Assessing the role of perceived stigma
Source: Psychooncology. 2018 Jul 3;27(9):2141–7. doi: 10.1002/pon.4779 (PMC6175243; doi:10.1002/pon.4779)
Supplement: Supplementary file 1 — Table S1 Self‐reported lung cancer characteristics (n=274)*. Table S2. Participants reported awareness, use and interest of one or more support service (n=274). Table S3. Participants’ reported likelihood of seeking help from people (n=274)*. [file PON-27-2141-s001.zip › TABLE S1_Stigma and help-seeking in lung cancer patients.docx]

**Table S1. Self-reported lung cancer characteristics (n=274)*.**

|  | **n (%)** |
| --- | --- |
| **Lung cancer type** | |
| Non-small cell lung cancer | 130 (51.6%) |
| Small cell lung cancer | 36 (14.3%) |
| Other (e.g. mesothelioma) | 14 (5.6%) |
| Don't know | 72 (28.6%) |
| **Cancer stage at diagnosis** | |
| Early | 72 (27.0%) |
| Advanced | 115 (43.1%) |
| Don't know | 80 (30.0%) |
| **Time since diagnosis** | |
| <4 weeks | 66 (24.3%) |
| 4-8 weeks | 97 (35.7%) |
| 8-12 weeks | 47 (17.3%) |
| 12-16 weeks | 62 (22.8%) |
| **Planned or completed treatment** | |
| Surgery | 55 (20.1%) |
| Chemotherapy | 184 (67.2%) |
| Radiotherapy | 104 (38.2%) |

* Number of observations varies due to missing data.
