# Supplementary material for: Help‐seeking behaviour in newly diagnosed lung cancer patients: Assessing the role of perceived stigma
Source: Psychooncology. 2018 Jul 3;27(9):2141–7. doi: 10.1002/pon.4779 (PMC6175243; doi:10.1002/pon.4779)
Supplement: Supplementary file 1 — Table S1 Self‐reported lung cancer characteristics (n=274)*. Table S2. Participants reported awareness, use and interest of one or more support service (n=274). Table S3. Participants’ reported likelihood of seeking help from people (n=274)*. [file PON-27-2141-s001.zip › TABLE S2_Stigma and help-seeking in lung cancer patients.docx]

**Table S2. Participants reported awareness, use and interest of one or more support service (n=274).**

|  | **Awareness,  ≥1 service** | **Used,  ≥1 service** | **Interested,  ≥1 service** |
| --- | --- | --- | --- |
|  | **n (%)** | **n (%)** | **n (%)** |
| **Overall** | 226 (85.0%)* | 197 (71.9%) | 149 (54.4%) |
| Emotional | 185 (81.9%) | 64 (32.5%) | 108 (72.3%) |
| Informational | 182 (80.5%) | 135 (68.5%) | 69 (43.6%) |
| Health professional | 197 (87.1%) | 137 (69.5%) | 101 (67.8%) |
| Practical | 209 (92.4%) | 128 (65.0%) | 126 (84.6%) |

*Missing, n=8
