# Supplementary material for: Help‐seeking behaviour in newly diagnosed lung cancer patients: Assessing the role of perceived stigma
Source: Psychooncology. 2018 Jul 3;27(9):2141–7. doi: 10.1002/pon.4779 (PMC6175243; doi:10.1002/pon.4779)
Supplement: Supplementary file 1 — Table S1 Self‐reported lung cancer characteristics (n=274)*. Table S2. Participants reported awareness, use and interest of one or more support service (n=274). Table S3. Participants’ reported likelihood of seeking help from people (n=274)*. [file PON-27-2141-s001.zip › TABLE S3_Stigma and help-seeking in lung cancer patients.docx]

**Table S3. Participants’ reported likelihood of seeking help from people (n=274)*.**

|  | **Likely to seek this person’s help** | **Unlikely to seek this person’s help** | **Unsure** |
| --- | --- | --- | --- |
|  | **n (%)** | **n (%)** | **n (%)** |
| Partner | 190 (81.9%) | 33 (14.2%) | 9 (3.9%) |
| Other relative/family | 213 (83.2%) | 21 (8.2%) | 22 (8.6%) |
| Friend/neighbour | 156 (61.4%) | 58 (22.8%) | 40 (15.7%) |
| Oncologist/treating clinician | 225 (89.3%) | 8 (3.2%) | 19 (7.5%) |
| Other health professional | 185 (72.8%) | 29 (11.4%) | 40 (15.7%) |
| Local general practitioner | 232 (91.0%) | 11 (4.3%) | 12 (4.7%) |
| Someone else | 65 (27.1%) | 133 (55.4%) | 42 (17.5%) |

* Number of observations varies due to missing data.
